# IGF axis and other factors in HPV-related and HPV-unrelated carcinogenesis (Review)

**DOI:** 10.3892/or.2014.3505

**Published:** 2014-09-19

**Authors:** JULIA DURZYŃSKA

**Affiliations:** Department of Molecular Virology, Institute of Experimental Biology, Faculty of Biology, Adam Mickiewicz University, 60-614 Poznań, Poland

**Keywords:** human papillomavirus, insulin-like growth factor, insulin-like growth factor receptor, somatotropic axis, signaling pathway, cervical cancer, therapy

## Abstract

The insulin-like growth factor (IGF) axis promotes the growth of cells, tissues and organs. IGF-1 is mainly produced in the liver but is also secreted from local tissues. In the circulation, IGF-1 is bound to insulin-like binding proteins (IGFBPs), and when released it activates the insulin-like growth factor receptor (IGF-1R). The signal is further transmitted by intracellular signaling pathways leading to gene expression that regulates, among others, cell proliferation and survival. This review presents the IGF axis in the context of cell transformation and cancer development. Aspects involving IGF-1 deficiency and protection from cancer are also briefly described. Furthermore, human papillomaviruses (HPVs) interplaying with IGF axis components in cervical cancer development are described. These small dsDNA viruses are divided into low-risk and high-risk HPVs with regard to the potency of their oncogenic actions; they mainly infect epithelial or mucosal cells. Special attention is drawn to expression of two major HPV oncogenes (E6 and E7) initiating and maintaining cervical carcinogenesis, which is a multistep and multifactorial process; therefore, involvement of additional factors such as mitochondrial DNA changes, sex hormones, retinoic and folic acids are also discussed. Finally, IGF axis components and HPV oncogenes as targets in anticancer treatment are presented which include IGF-1R downregulation, RNA interference and anti-HPV therapeutic vaccines. The review concludes that despite an enormous advancement in research on IGF and HPV-related cancers, more molecular studies and clinical trials are needed before commercialized therapies are widely available for oncology patients.

## 1. Introduction

**Figure 1:**
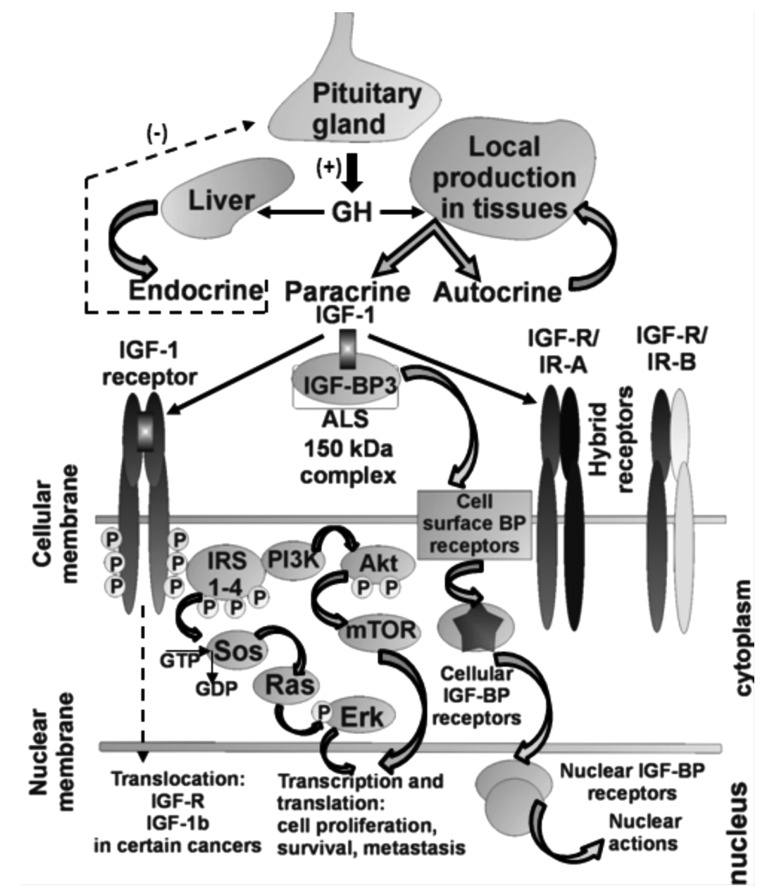
Schematic presentation of IGF-1 axis actions. For simplicity only major IGFBP is shown (IGFBP3) forming a 150-kDa complex with the IGF-1 ligand and ALS (acid labile subunit). Notably, all 3 components of the IGF-1 axis can be translocated to the nucleus: i) IGF-1 (B isoform containing a nuclear localization signal at C-terminus of the E peptide, precise function unknown); ii) IGF-1R as demonstrated in renal cancer, probably involved in transcription regulation; iii) IGFBPs can be translocated to the nucleus via their nuclear receptors and have functions independent of IGF-1 and IGF-1R. Hybrid receptors (IGF-R/IR-A and IGF-R/IR-B) are also activated with lower affinity by IGF-1 ligand as compared to IGF-1R.

The IGF axis stimulates the growth and proliferation and involves many key molecules such as insulin-like growth factor (IGF)-1 and IGF-2, their transmembrane receptors (IGF-1R and IGF-2R, respectively), the IGF-binding proteins (IGFBPs) and intracellular signaling proteins such as the insulin receptor substrate (IRS) family, Akt and many others. Initially, it was believed that all IGF-1, which is both a growth hormone and a tissue growth factor of 70-amino acids in length, originated in the liver and was transported by an endocrine mode to sites of action. At present, it is recognized that IGF-1 is also produced in other organs where paracrine and autocrine mechanisms are engaged (1). Alternative splicing (AS) of the *IGF-1* gene results in multiple isoforms that retain the identical sequence of mature IGF-1, but also give rise to divergent C-terminal E-peptides. The peptides may modulate the actions, stability, or bioavailability of IGF-1, or they may have independent activity. Six different splice forms can be produced; from either of the two different promoters P1 and P2 three isoforms, IGF-1A, IGF-1B and IGF-1C, are transcribed (2). Recent data indicate that the entire IGF network is even more complicated as in some tissues more than one form of IGF-1A can be active. In mice, three forms in different proportions have been detected in muscle: mature IGF-1, pro-IGF-1 (C extension is not cleaved) and glycosylated pro-IGF-1 (C-extension has bound sugars residues and is not cleaved) (3). Furthermore, it has been shown in two independent studies that human Eb-peptide cleaved form human pre-pro-IGF1b, which is 77 amino acids long, localized to the nuclei of transfected cells and may have IGF-1 independent mitogenic and bioactive properties (4–6). Notably, a 10-fold decrease in the IGF-1B transcript level was observed (7), and a downshift of the IGF-1B content in favor of the IGF-1A isoform was reported when non-tumor tissue and colorectal cancer cells were analyzed (8). On the other hand, an increase in IGF-1B and decrease in IGF-1A expression were found in cervical cancer and control cells, respectively (9). It is now clear that it is important to understand, not only the overall IGF expression level, but also the entire IGF isoform profile assuring a whole new level of IGF-1 activity regulation in local tissues linked to the presence of different IGF forms and the presence of different forms of the same isoform (glycosylated pro-IGF-1A) (Fig. 1).

## 2. IGF axis and cancer

Recently, accumulating evidence indicates that the IGF axis is involved in human cancer progression (10). IGF-1 signaling can contribute to each stage of cancer progression: malignant transformation, tumor growth, local invasion and distant metastases, and resistance to treatment. In addition to direct contributions to each of these stages, IGF-1 may promote cancer indirectly, through interactions with oncogenes and tumor supressors, with other hormones (particularly sex steroids in breast and prostate cancers) and with IGFBPs (11). The findings of another study suggest that elevated IGF-1 levels may be implicated in the development of ovarian cancer, diagnosed before age 55 years (12). Whereas in colorectal carcinoma, the local expression levels of total IGF-1 mRNA and all splicing isoforms of IGF-1 mRNA were decreased as compared to normal colon tissues. The results of this study suggest an increased regenerative potential in normal colon tissues which, at least partially, is linked to an elevated expression of total IGF-1 mRNA and its isoform A (8). An important clue to the essential role of the IGF-1R in cellular function was uncovered by Sell and co-workers who reported that IGF-1 signaling is an absolute requirement for viral transformation of cells (13). Numerous studies performed over the last 20 years have suggested that transformed cells express the IGF-1R at higher levels than normal cells. However, a decade ago the molecular mechanisms by which IGF-1R gene expression is increased in tumors remained largely unidentified (14). Further *in vitro* studies have demonstrated that a functioning IGF-1R is necessary for cell transformation by many viral and cellular oncogenes and appears to be important in expressing the genes that regulate the cell cycle, cell survival, motility, attachment and metastasis (15,16). Cell surface IGF-1R translocates to the nucleus following clathrin-mediated endocytosis, regulated by IGF levels. The IGF-1R is unusual among transmembrane receptors that undergo nuclear import, in that both α and β subunits traffic to the nucleus. Nuclear IGF-1R is phosphorylated in response to ligands, and undergoes IGF-induced interaction with chromatin, suggesting direct engagement in transcriptional regulation. Nuclear IGF-1R is detectable in primary renal cancer cells, formalin-fixed tumors, preinvasive lesions in the breast, and non-malignant tissues characterized by a high proliferation rate. In clear cell renal cancer, nuclear IGF-1R is associated with adverse prognosis. These findings suggest that IGF-1R nuclear import has biological significance, and may contribute directly to IGF-1R function (Fig. 1) (17). It has been pointed out that the IGF-1R alone does not mediate growth and transforming activities, but rather the pathway itself, which is administered by IRS-1, signals to growth promoting and anti-apoptotic pathways. It is clear that IRS-1 is a key hub overseeing downstream signaling actions of the IGF-1R, and IRS-1 could be referred to as an antitumor suppressor acting as an anti-p53 protein (18,19). The role of the IGF-1R in the progression of epithelial tumors that are more prevalent in adults is likely to be more complex (20); however, the prevailing notion that IGF-1R is routinely overexpressed in transformed cells is somewhat of an overgeneralization (14).

## 3. IGF-1 deficiency and protection from cancer

Significantly, IGF-1 deficiency is a major contributing factor in lifespan prolongation especially in females and in protection from cancer (21,22). It has been extensively studied in individuals with Laron syndrome (LS) who have an inactive GH receptor and IGF-1 deficiency leading to dwarfism; a recessively inherited syndrome caused by deletions or mutations in the GH receptor or post-receptor pathways (21,23). The cohort of LS patients, treated or not treated by recombinant IGF-1, appears to be protected not only from cancer but also from diabetes, whereas taller stature is now regarded as a risk for several types of cancer (24). Growth disorders are multifactor, complex phenomena with often-unknown etiology, and in-depth large-scale pooled next-generation sequencing is used for molecular diagnosis (25). It has been demonstrated that GH, GHR, IGF-1 and IGF1-R coding sequences may be altered in growth disorders (26,27); however, these sequences are not changed in the genome of children with short stature (28,29) and the search for other defective genetic backgrounds related to the IGF-1 axis should be considered. In order to further clarify the relationship between GH/IGF-1 and cancer more studies are needed.

## 4. IGF axis and viruses in cancer

Many studies indicate that the IGF axis and viruses can combine their actions in cellular transformation leading to cancer. Several components of the IGF signaling axis, such as IGF-1, IGF-2 and IGF-1R, are deregulated during HCV-related human hepatocellular carcinoma (HCC). Only a few investigations have focused on hepatic expression of IGFs and their receptors at different stages of chronic hepatitis C infection. The studies demonstrated an increased IGF-1R synthesis, aberrant IGF-2 expression (decreased/increased), and decreased synthesis of IGF-1 as events in human hepatocarcinogenesis. Recognition of the role played by HCV in different splicing profiles of the *IGF-1* gene in the progres sion of chronic hepatitis C will require further study. A better understanding of the interactions between HCV protein and IGF axis component will facilitate the development of novel approaches to prognose and to treat virus-related HCC (30).

Large T-antigen from the human John Cunningham polyomavirus (JCV T-antigen), also present in the SV-40 virus (both viruses belong to *Polyomaviridae*), inhibits homologous recombination directed DNA repair (HRR), which results in an accumulation of mutations. Following T-antigen-mediated nuclear translocation, IRS-1 binds Rad51 at the site of damaged DNA. This T-antigen-mediated inhibition of HRR does not function in cells lacking IRS-1, and can be reproduced in the absence of T-antigen by IRS-1 with an artificial nuclear localization signal (31). The interplay described between the IGF-1R signaling system and JCV T-antigen in the process of DNA repair could be relevant, since nearly 90% of the human population is seropositive for JC virus, the JCV T-antigen transforms cells *in vitro*, the JCV T-antigen is tumorigenic in experimental animals, and the presence of the JC virus has been noted in an increasing number of biopsies of human cancer (32). The family of *Papovaviridae* was taxonomically split into the *Papillomaviridae* (HPV) and the *Polyomaviridae* (JCV). John Cunningham virus expresses a T-antigen that causes malignant transformation through development of aneuploidy and interaction with some of the same regulatory proteins as HPV (33).

## 5. HPV in cervical cancer

Human papillomaviruses (HPVs) constitute a heterogeneous group of viruses from the *Papillomaviridae* family. They are double-stranded circular DNA viruses with an icosahedral capsid and are able to infect epithelial cells. An HPV phylo-genetic tree has been designed based on the homologous nucleotide sequence of the major capsid protein L1 that groups the different HPV types into genera: α, β, γ, δ, μ and others (34). The HPV genome is approximately 8 kb in length and is divided into three regions, the non-coding long control region (LCR), and the coding early (E) and late (L) regions. The viral genome encodes six early (E1, E2, E4, E5, E6, E7) and two late proteins (L1 and L2). The transcription of early and late genes is controlled by the LCR. The viral proteins are translated from polycistronic mRNAs containing overlapping reading frames (35). Papillomaviruses have been extensively studied and more than 100 different types have been identified (36). The association between HPV and human cancer was first proposed more than three decades ago by Herald zur Hausen, and since then additional studies have fully demonstrated the direct role of HPV infection in the development of several human cancers (37). Depending on their potential to induce carcinogenesis HPVs have been divided into low-risk (HPV-6 and -11) and high-risk (HPV-16 and -18) (38). HPVs can also be held responsible for anogenital, head and neck, skin and other types of cancer (39). The mucosal HPV types preferentially infect the cervical transformation zone, which is the junction point of the endocervix columnar cells and the ectocervix stratified squamous epithelial cells (40). Many HPV types cause only productive lesions following infection and are not associated with human cancers. In such lesions, the expression of viral gene products is carefully regulated, with viral proteins being produced at defined times and at regulated levels as the infected cell migrates towards the epithelial surface. The pattern of viral gene expression in low-grade cervical lesions resembles that observed in productive warts caused by other HPV types. High-grade neoplasia represents an abortive infection in which viral gene expression becomes deregulated, and the normal life cycle of the virus cannot be completed (41). As is typical of viruses that co-evolve with their hosts, many PVs produce only chronic, inapparent infections and produce virions from the surface of infected epithelium without apparent detriment to the host (42). The paradox is that the infection with oncogenic types of HPV is very common and most of these infections go unnoticed. Malignancy is a rare outcome of a common HPV infection (43). However, not all HPV types use the same strategy and it appears that several of the α PVs, in particular, have acquired immunoevasion strategies that allow them to cause persistent visible papillomas (42).

HPV genomes replicate episomally in host cells, but HPV DNA is frequently found to be integrated into chromosomes in cervical cancer. The timing of viral integration appears to correspond to the development of high-grade cervical intraepithelial neoplasia (CIN) as a consequence of high-level expression of E6 and E7 (44). Vinokurova and co-workers suggest that HPV integration is not an essential event in cervical carcinogenesis. This integration of oncogenic HPV genomes in cervical lesions is a consequence rather than the cause of chromosomal instability induced by deregulated high-risk papillomaviruses (HR-HPVs) E6–E7 oncogene expression. The integration frequency of various HPV types is strongly correlated with the age at diagnosis of cancer, suggesting that the malignant potential of the various HR-HPV types is reflected by their integration frequency in invasive cervical carcinomas (45). Integration occurs near fragile sites in the human genome and results in termination of the viral cycle as large portions of the genome are disrupted and therefore it becomes functionally inactive (46). The alternative mechanism by-passing viral integration and E2 gene disruption which enables pure episomal HPV genomes to maintain an upregulated expression of E6 and E7 oncogenes is methylation of E2 binding sites at the promoter region of HPV-16 (47).

E6 and E7 have various biological activities in addition to inactivation of the major tumor suppressors, p53 and pRB, respectively (48). It has been suggested that for a lesion to be maintained, the virus must infect an epithelial stem cell (49). It is generally thought that the viral E1 and E2 proteins are expressed in order to maintain the viral DNA as an episome (50) and to facilitate the correct segregation of genomes during cell division (51). Expression of E6 and E7 in the lower epithelial layers drives cells into the S-phase, which creates an environment that is conducive for viral genome replication and cell proliferation (52). E6 and E7 oncoproteins cooperate in cellular transformation and evasion of the immune system (40). The PDZ domain-binding motif of E6 [X-(S/T)-X-(V/L/I) where X is any residue, S/T is serine or threonine and V/L/I is valine, leucine or isoleucine] appears critical for its transforming activity in cultured cells and tumorigenicity in xenograft experiments. In contrast, none of the low-risk HPV E6 proteins have this motif (53). Moreover, a study presented by Sun *et al* suggests that trapping of p53 in the cytosol by HPV-11E6 results in apoptosis and represents a novel mechanism to explain why low-risk HPV infection does not result in malignant transformation; this explanation remains tentative, and awaits further testing (54). The best characterized high-risk HPV-16 E6 activity is its ability to induce degradation of the tumor-suppressor protein p53 via the ubiquitin/proteasome pathway. This cellular protein is a transcription factor that can trigger cell cycle arrest or apoptosis in response to a large variety of cellular stresses, such as hypoxia or DNA damages (55). E6 targeting PDZ domain-containing proteins such as hDlg, hScribble and p53, further supplemented by induction of the catalytic subunit of telomerase reverse transcriptase (hTERT) contributes to cellular immortalization. Targeting pRb and its family members for degradation by E7 oncoprotein constitutes a major step in tumorigenesis. Both E6 and E7 bind and inactivate several transcription factors involved in the immune response, e.g. interferon regulatory factors (IRFs). This serves to avoid immune-based destruction of HPV-containing tumors while acquiring invasive potential through modulation of other pathways (56). The HPV E7 protein shares functional similarities with such proteins as adenovirus E1A and SV40 large tumor antigen. In the HPV viral life cycle, E7 disrupts the intimate association between cellular differentiation and proliferation in normal epithelium, allowing for viral replication in cells that would no longer be in the dividing population (57). HPV-16 is the most prevalent genotype in cervical carcinoma and is also the most frequently detected HPV types in head and neck squamous cell carcinoma (HNSCCs). It is also interesting to note that HPV-associated oropharyngal squamous cell cancers have a better prognosis than HPV-negative tumors (58). As determined by PCR, HPV-16 DNA is present in ~56% of SCCs of the cervix. HPV-18 is the second most common HPV type associated with cervical adenocarcinoma, causing 37–41% of SCCs (59). Little is known concerning the apparent absence of HPV infections in the gastrointestinal epithelia (60). None of the individual reports claiming the presence of anogenital HPVs in cancers of the esophagus, prostate, bladder, lung, demonstrated a consistent association of these viruses with cancer of these respective sites (39).

## 6. IGF axis and HPV-related cervical cancer

The association between HPV infections and IGF levels has not been extensively explored in cervical neoplasia; nevertheless, a growing number of studies have recently demonstrated an association between serum levels of IGFs and IGFBP-3 and increased risk for various cancers. For example, it was shown that the plasma IGF-1 level and IGF-1/IGFBP-3 molar ratio were significantly associated with CIN, but had no significant association with cervical cancer. However, it is not clear whether increased plasma levels of IGF-1 and IGF-1/IGFBP-3 were the cause or the result of CIN. Despite all these significant associations observed, the results of this study showed no relationship between IGF levels and HPV infection status (61). Another much more prospective study was one of the first to demonstrate a relationship between serum levels of IGF-1 and precancerous squamous intraepithelial lesions (SILs). Individuals with either high-grade (HSILs) or low-grade SILs (LSILs) exhibited significantly higher serum levels of IGF-1, IGFBP-3 and IGF-1/IGFBP-3 molar ratio than did control subjects. Furthermore, there was a dose-dependent relationship between risk of SILs and levels of IGF-1. After adjustment for IGF-1, no relationship was evident between the IGFBP-3 level and risk of SILs (62). On the other hand, the results from another study of preoperative serum total IGF-1 or IGFBP-3 levels failed to predict cervical cancer mortality and recurrence. It is difficult to explain why increased serum IGF-1 level may have a protective effect on the risk of cervical cancer observed, whereas the unfavorable effect of serum IGF-1 is addressed in certain sex hormone-related cancers, such as prostate or breast cancer (63). The authors of the study concluded that more candidates should be enrolled in further studies to realize the differences in serum levels of IGF-1 between normal individuals, patients with precancer lesions and cervical cancer (63). In concordance with these observations, another more prospective study demonstrated that increased levels of IGF-1 are associated with reduced risk of HCIN. High IGF-1 concentration was associated with a reduced risk of being positive for HPV-16 and -18. Levels of IGFBP-3 were not associated with the risk of HCIN or being HPV positive among controls. Yet again, it is unclear why increased levels of IGF-1 would have a protective effect on the risk of cervical cancer precursors and that the protection would be stronger among younger women. One possible explanation is that IGF-1 decreases a woman’s risk of high-grade cervical intraepithelial neoplasia (HCIN) by decreasing her risk of being positive for HPV-16 and/or -18, perhaps via increased turnover of the cervical epithelium, thus reducing the duration of infections (64). Additionally, it has been shown that a lower serum IGF-1 level is correlated to increased risk of cervical cancer (65), and that the differential expression of IGF components in controls, LSILs, HSILs and cervical cancer, could be related with the carcinogenic process in cervical epithelium and could be a potential marker for progression (66). The same research group demonstrated that cervical cancer cell lines, positive and negative for HPV, differ in the type of insulin and IGF-1 receptors expressed, while SiHa cells expressed IGF-1R, IR-A and IR-B and IR/IGF-1R hybrid receptors, C33a cells expressed the IR-A only (67). Results showed that median protein levels of IGF-2 were significantly lower in cervical cancer cases vs. controls and significantly higher values of IGFBP-3 were found in HSIL vs. controls, and were not affected by HR-HPV infection. Meanwhile no significant differences were observed in IGFBP-3 levels between LSILs or cervical cancer as compared to controls. These significant data suggest that the progression to cervical cancer is associated with alterations in the IGF system and is not affected by HR-HPV infection. More studies are needed to understand the possible role of IGFBP-3 in cervical carcinogenesis (68).

It has been observed that the growth and invasiveness of cervical cancer cells are dose-dependently stimulated by IGF-1, whereas the growth and invasiveness of normal cervical epithelial cells are not. It was pointed out that IGF-1, acting through IGF-1R, interacted with α_v_β_3_ integrin in cervical cancer cell invasiveness and proliferation (69). Transformed cells that overexpress the IGF-1R may subvert growth regulation by minimizing their dependency on additional growth factors. Data demonstrating that the IGF-1R is overexpressed in both primary cervical tumor cells and cervical cell lines are consistent with this concept (70). Furthermore, in a study by Kuramoto *et al* the expression levels of IGF-1R were significantly higher in CIN and invasive cancer specimens. IGF-1R was overexpressed in HPV-positive cervical cancer cell lines in comparison to ovarian cancer cell lines and HPV-negative cervical cell line C33A. Phosphorylation of IGF-1R was promoted in all CIN and invasive cancer and its intensity was related to the promotion of lesions (71). In a retrospective study of patients with early-stage cervical cancer it was shown that high overexpression of IGF-1R is an independent predictor of cervical cancer death and recurrence (63). On the other hand, low levels of IGFBP-3 and IGF-1R mRNA in cervical scrapes were found to be associated with progression to cervical cancer. Low levels of IGF-1R mRNA were already reported in certain types of cancer, for example breast cancer (72).

The IGF binding proteins (IGFBPs) represent the third important component of the IGF system, after IGF-1 and IGF-1R, consisting of a class of six soluble secretory proteins. They represent a unique class of naturally occurring IGF antagonists that bind and sequester IGF-1 and IGF-2, limiting their access to the IGF-1R (73). Elevated IGFBP-3 levels may have a protective function in ovarian cancer occurrence (74). In addition, IGFBP-3 is a proapoptotic agent and has been shown to act through mechanism(s) independent of IGFs (75). In contrast to early-passage cells, late-passage cells were found to secrete IGFBP-3 and showed an increased response to IGF-1 as determined by the IGF-1R and insulin receptor substrate (IRS) phopshorylation. Thus, the increased responsiveness of HPV-immortalized cells to IGF-1 could potentially contribute to their *in vivo* growth, where IGF-1 is produced by surrounding stromal cells (76). The induction of IGFBP-3 during immortalization is somewhat surprising given previous knowledge of its regulated expression and activity. Additionally, strong transcriptional activators of the IGFBP-3 gene must exist and appear in late-passage HPV-16 E6/E7 cervical cells. *In situ* hybridization results showing overexpression of IGFBP-3 mRNA in HSIL patient samples supports the findings of IGFBP-3 upregulation in immortalized cervical cells (77). In another study, the influence of circulating IGF-1 levels on the natural history of oncogenic HPV was prospectively assessed. Women with high serum IGFBP-3 levels had significantly lower rates of incident oncogenic HPV detection, and a lower incidence of oncogenic HPV-positive SIL, than woman with low serum IGFBP-3 levels. The IGF1/IGFBP-3 molar ratio, in contrast, was positively associated with persistence of oncogenic HPV infection. Thus, high IGFBP-3 levels could lead to less replication and/or greater loss of oncogenic HPV-infected cells. Although in the pilot investigation none of the additional associations of oncogenic HPV with IGF-1 or IGFBP-3 were statistically significant, they were fairly similar to the above: total IGF-1 had a nonsignificantly positive association with prevalence of oncogenic HPV and oncogenic HPV-positve SIL, whereas high IGFBP-3 had a nonsignificant inverse association with these same endpoints. There was one inverse association with IGF-1 in the study: high total IGF-1 was associated with a lower (not a higher) prevalence of nononcogenic HPV (78). While it is generally assumed that properties of HPV E7 depend on its interaction with regulators of the cell cycle, E7 can also directly bind IGFBP-3, the product of a p53-inducible gene that is overexpressed in senescent cells. IGFBP-3 can suppress cell proliferation and induce apoptosis; IGFBP-3-mediated apoptosis is inhibited by E7, which binds to IGFBP-3 and triggers its proteolytic cleavage (79). As it is generally assumed that elevated IGFBP-3 level has a protective function in cancer, it was reported that serum levels of IGFBP-3 in patients with cervical cancer are significantly lower than levels in controls, and they revert to normal following therapy (80).

While comparing data from different studies, a distinction is needed between results based on an extensive body of evidence in well-conducted prospective studies and those in small studies with weak cross-sectional design is important (Table I). It is certain that most of the studies showed a strong association of IGFs with the HPV status and cancer risk (61–64,78,80). It was also indicated in an important review by Pollak *et al* that increasing IGF-1 levels are associated with an increased risk of cancer since somatic cells of individuals with higher levels of IGF-1 may show slightly higher proliferation rates and have a slightly increased chance of survival in the presence of genetic damage because of the antiapoptotic effects of IGF-1 (model of stepwise accumulation of genetic damage leading to carcinogenesis) (1). The only unclear aspect of the association being discussed is that some researchers claim it is positive (61,62,81) and others claim the contrary is the case (64–66) and even though there is a stronger body of evidence supporting the former, the latter should not be discarded.

## 7. Other factors in cervical cancer

Development of cervical cancer affects a small percentage of HR-HPV-infected women and often takes decades after infection, suggesting that HR-HPV is a necessary but not sufficient cause of cervical cancer (81). Thus, other cofactors are necessary for progression from cervical HR-HPV infection to cancer. These factors include long-term use of hormonal contraceptives, multiparity, smoking, as well as micronutrient depletion and in particular retinoid deficiency, which alters epithelial differentiation, cellular growth and apoptosis of malignant cells. Therefore, early detection of HR-HPV and management of precancerous lesions together with a profound understanding of additional risk factors could be a strategy to avoid this disease (82). Chronic estrogen exposure is a key factor for the development of this disease. E6 oncogene was found to synergize with estrogen to induce cervical cancer after 9 months, indicating that E6 has a weaker but detectable oncogenic potential in the reproductive tract compared with the E7 oncogene (83). Estrogens upregulate HPV E6/E7 oncogene expression, stimulate cell proliferation, inhibit apoptosis and their metabolites cause DNA damage. On the other hand, retinoid deficiency is implicated in cervical squamous metaplasia and the decrease in retinoic acid receptor β (RARβ2) expression promotes AP1-dependent cellular proliferation. Synergistic activation of cell proliferation by viral oncoproteins, estrogen receptor signaling, inhibition of RARβ2 expression and nutritional status factors may conspire to support and promote neoplastic progression and cervical cancer (82). While the uterine cervix is highly responsive to estrogen, the role of estrogen in cervical cancer, which is strongly associated with HPV infections, is poorly understood. Estrogen and estrogen receptor α (ERα) are required for cervical carcinogenesis, and cervical cancer is often positive for ERα, although its functionality in this cancer has yet to be demonstrated (84). Tumors arising in HPV-16 transgenic mice treated with estrogen for 9 months were greatly increased in size compared with tumors developing after 6 months of estrogen treatment. It can be concluded that estrogen plays a critical role not only in the genesis of cervical cancer but also in its persistence and continued development in this mouse model (85). A transgenic mouse model expressing HPV oncogenes E6 and/or E7 has proven useful to study a mechanism of hormone actions in the context of this common malignancy. ERα is known to upregulate expression of the progesterone receptor, which, on activation by its ligands, either promotes or inhibits carcinogenesis, depending on the tissue context. These results provide the first experimental evidence that supports the hypothesis that progesterone signaling has an inhibitory effect on cervical carcinogenesis *in vivo* (86). With regard to apoptosis, it has been suggested that the stimulatory effects of 17β-estradiol on E2 and E7-induced cell death are mediated by 16α-hydroxy-estrone in HeLa cells (87). It was also shown that cervical malignant cells tend to lose the ERα but maintain the ERβ actively expressed. Loss of expression of ERα in neoplastic tissue suggests that the estrogenic effects could be regulated through the ERβ in human neoplastic cervical tissue (88).

It has been reported that HPV-18 E6 and E7 proteins directly interact with nuclear receptors (NRs) such as thyroid receptor (TR), androgen receptor (AR) and ER through a hormone-independent mechanism (89). HPV-18 E6 protein was found to generally enhance the reporter activities of these three NRs. In contrast, HPV-18 E7 protein repressed the reporter activities of these three NRs either in the absence or presence of cognate ligands. However, in HeLa cells (compared with HEK 293 cells), in the absence of an appropriate ligand, no coregulatory effect on AR, ER and TR was detected, whereas these NR activities increased up to 7-fold in the presence of hormone (89). Folic acid supplementation might be useful in maintaining cervical health as it upregulates IGFBP-3 (90). Other findings are in agreement with this observation and indicate that it may be important to improve the folate status in HR-HPV-infected women and that folate supplementation should be assessed as a viable option for reducing the risk of developing CIN ≥2 in women exposed to HR-HPV, especially HPV-16 (91). It has been reported that higher circulating concentrations of folate are independently associated with a lower likelihood of becoming positive for HR-HPVs and of having a persistent HR-HPV infection and a greater likelihood of becoming HR-HPV-negative (92).

**Figure 2:**
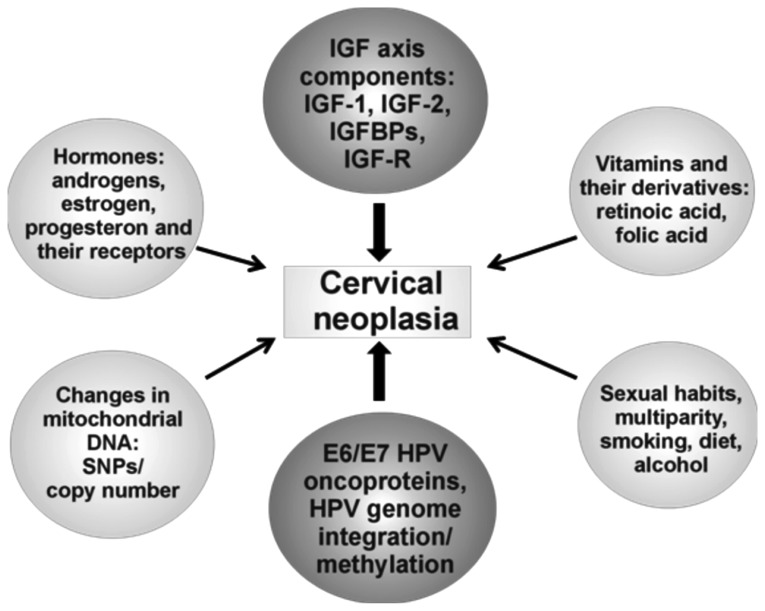
Schematic representation of cervical neoplasia as a multifactor process. A multitude of factors with oncogenic activities are gathered in six groups. For clarity, the potential or known interactions between these groups are not indicated as they are discussed in the text. Special attention is drawn to the IGF axis and HPV oncogenes.

Alterations in mtDNA both qualitatively (by mutations) and quantitatively (by mtDNA copy number) are associated with cervical cancer development. High levels of mtDNA copy with a 4.997 bp deletion in LSIL cells can be associated with the susceptibility of cells to an HPV-persistent infection and cervical cancer development (93). The copy number of mtDNA in cases which carried a D-loop mutation was significantly higher than that of the negative cases (P<0.05). These results suggest that the mtDNA D-loop in low-grade squamous cell carcinoma (LSCC) is an unstable region with a high frequency of somatic mutations and polymorphisms. Together with the increase in mtDNA copy number, these factors may play a role in carcinogenesis of the larynx (94). Mutations of mtDNA in breast cancer occur both within and outside of the D-loop, although the mutation rate in the D-loop is more than 7-fold higher than in coding areas. There have been 26 new mutation loci identified (25 in regions sequenced by others, one in an area not sequenced). The high frequency of mtDNA mutations at polymorphic loci requires further investigation (Fig. 2) (95).

## 8. Cancer therapy: IGF and HPV targeting

The IGF axis has emerged as a meaningful therapeutic target for oncology drug development and is strongly supported by preclinical studies and promising results from early phase clinical trials. The 3 major classes of IGF-targeted therapeutic compounds [i.e., IGF-1R-specific monoclonal antibodies (mAbs), small-molecule tyrosine kinaze inhibitors (TKIs) targeting IGF-1R and IR kinase domains, and finally, an IGF-1 and IGF-2 Ligand-neutralizing mAbs)] differ in the range of target inhibition based on their ability to block activation of IGF-1R, IGF-1R/IR-A hybrid and IR-A. They also exhibit different safety profiles, most notably with respect to modulation of glucose metabolism, as well as through changes in circulating levels of IGF, insulin, and growth hormone (96). By 2010 there was a list of over 30 drugs under evaluation as single agents or in combination therapies (73). Recently, targeting therapy with the IGF-1R antibody has rapidly developed (97,98). The treatment of blocking IGF-1R with antibodies markedly decreased IGF-1R phosphorylation and downstream activation of Akt and Erk1/2, hereby inhibiting tumor growth (96). IGF-1R has been detected in many studied tumors, regardless of differentiation or proof of EBV or HPV integration into the genome. These results suggest that IGF-1R expression in these tumors is capable of transmitting mitogenic signals to the neoplastic cells (99). The IGF-1R antibodies appear to have a favorable safety profile and have been demonstrated to reduce IGF-1R signaling in patients. Concerning the IGF-1R tyrosine kinase inhibitors, the first published data from clinical trials are still awaited. Some phase II and III trials have been suspended or terminated, because of the lack of efficacy of the antibodies. The identification of predictive biomarkers is of crucial importance for the further development of anticancer therapies based on anti-IGF-1R agents (100). However, the results of targeting the IGF-1R with specific antibodies for ‘financially attractive tumors’ (breast, colon, prostate, lung cancers and others) have been unsatisfactory. In addition, it should be remembered that blocking IGF-1R with therapeutic antibodies can lead to metabolic toxicity (101). Effective targeting of the IGF system may require a customized approach in which tumor profiling guides the selection of the appropriate drugs (102). In the next few years, exciting clinical trials and translational research will provide information and explanations in order to identify biomarkers for anti-IGF-1R treatments, thus allowing the targeting of populations that will most benefit from anti-IGF-1R mAbs and combined treatments (103). Indeed, there is very little to encourage the further use of targeting the IGF-1R as a single agent in treatment of human cancer, except in a few, relatively rare tumors. There is more hope in multi-drug therapies (104) and multiple challenges are still ahead, including the multiplicity of potential cancer indications and drug combinations, as well as the need of biomarkers for resistance and sensitivity (100) and for the time being demonstration of meaningful clinical benefit remains elusive (96).

Tumorigenesis in nude mice was found to be highly inhibited in HeLa S3 and SiHa clones transfected with the IGF-1R antisense RNA. These results indicate that downregulation of IGF-1R can reverse the transformed phenotype of human cervical cancer cells, even when harboring malignant type HPVs (105). HPV-associated cancers are prime candidates for the development of RNA interference-based therapeutic approaches (106). Increasing evidence has shown that microRNAs are commonly deregulated in human malignant cancers, including cervical cancer (107) but the role of microRNA (miR)-497 in human cervical cancer still remains unclear. Recently, miR-497 was demonstrated to bind to the 3′ untranslated regions of IGF-1R mRNA, and upregulation of miR-497 downregulated IGF-1R protein expression. Further investigation showed that small interfering RNA-mediated IGF-1R knockdown could mimic the effect of enforced miR-497 expression on the malignant phenotypes of cervical cancer cells (108). Furthermore, 12 highly differentially regulated miRNAs, which distinguished high-grade CIN specimens from normal cervical epithelium have been identified. Target prediction analysis revealed that these deregulated miRNAs mainly control apoptosis signaling pathways and cell cycle regulation. These findings contribute to understanding the role of miRNAs in the pathogenesis and progression of cervical neoplasm at the molecular level (109). Previous studies have demonstrated that small interference RNAs (siRNAs) can suppress HPV6 and/or E7 expression in various cancer cell lines (110,111). The advantage of using a lentiviral delivery system compared to synthetic and vector-borne siRNA, used in previously mentioned studies, is the ability to stably transduce dividing and non-dividing cells with relatively high efficiency (112). Data by Gu and co-workers collectively suggest that lentiviral delivery is an effective way to achieve stable suppression of E6/E7 oncogene expression and induce inhibition of tumor growth both *in vitro* and *in vivo*. They demonstrated a high specificity for LV-18E6-1, which kills only HeLa cells but not other cervical carcinoma cells without HPV-18 E6 such as C33A and SiHa. These results encourage further investigation of this form of RNA interference as a promising treatment for cervical cancer (113). There has been mild success in treating different diseases with the use of lentiviral vectors (114). Similar results were obtained in another study. LV-shRNA specific to HPV-16 oncogenes, targeting the promoter and the E6-transcript, effectively knocked down E6 and E7 expression, along with accumulation of p53 and pRB protein, resulting in markedly reduced abilities of proliferation and invasiveness of cervical cancer cells *in vitro*. These findings may provide important evidence for the application of LV-shRNA targeting HR-HPV key oncogenes, as a new treatment strategy, in cervical and other HPV-associated cancer therapy (115). HPV pseudovirions encoding shRNA may provide another means of cervical cancer therapy, and using shRNA/HPV pseudovirions appears to be a more promising strategy than using siRNA alone. The presence of L1 capsids can also induce local immunization against L1 neutralizing epitopes, thus conferring protection in cases of HPV reinfection after the death of E7-expressing cells. These pseudovirions represent a new step in designing rational molecular cancer therapy using RNA interference, at the same time, offering protection against reinfection by the causative agent of cervical cancer (116). Another new active targeted immunotherapeutic has been evaluated, Modified Vaccinia Virus Ankara (MVA) vector, containing the E1 sequence of HPV-16, aimed at inducing cellular immune responses with potential to help and clear persistent HPV-16-related infection. It has been shown that multiple injections of MVA-E1 allowed sustained HPV-16E1-specific cellular immune responses in vaccinated mice and had no impact on the exhaustion phenotype of the generated HPV-16E1-specific CD8^+^ T cells, but led to the differentiation of multi-functional effector T cells with high cytotoxic capacity. This study provides proof of concept that a MVA expressing HPV-16E1 can induce robust and long lasting E1-specific responses, and further development of this candidate is warranted (117).

The availability of prophylactic HPV vaccines has provided powerful tools for primary prevention of cervical cancer and other HPV-associated diseases. By the beginning of 2012, the HPV vaccine had been introduced into national immunization programs in at least 40 countries (118). Prophylactic vaccines induce neutralizing antibodies against HPV L1 structural proteins, which are associated with protection from HPV infection. However, therapeutic vaccines induce cytotoxic T lymphocyte (CTL) responses to HPV early regulatory proteins, possibly leading to eradication of CIN, cervical cancer and other HPV-associated diseases. The antibodies neutralize infectious HPV particles, while CTLs recognize and kill HPV-infected epithelial cells and HPV-associated cancer cells (119). Indeed, E6 and E7 are often the only viral genes that continue to be expressed in cancerous cells (120), thus they represent ideal targets for immunotherapy of cervical cancer. Accordingly, numerous methodologies to elicit strong anti-E6/E7 cellular immunity have been explored including peptide immunization (121), DNA immunization (122), immunization with recombinant, E7-expressing Vaccinia virus (117), adenovirus (123), pathogenic bacteria (124), E7-pulsed dendritic cells (125) or E7-containing virus-like particles (VLPs) (126). Although a number of approaches in therapy of cervical cancer have been developed, none has yet advanced for commercial use. These approaches are summarized in Table II.

## 9. Conclusions

This review discusses cervical carcinogenesis as a multifactor and multistep process. Although substantial progress has been made in cancer therapies focused on blocking HPV oncoproteins and IGF axis components, mainly IGF-R, there are many molecular and clinical studies in progress aimed at the development of a more effective treatment of cervical neoplasia. Multidrug combinatorial therapies will greatly help overcome the difficulties described in the present review.

## Figures and Tables

**Table I:** Summary of the possible correlations between IGF axis components, HPV status and cervical cancerogenesis.

Risk/incidence of neoplastic change/cancer (increased↑/decreased↓/no associationΔ)	Correlation with serum or local tissue level (higher↑/lower↓/no changeΔ)	Correlation with HPV status (positive↑/negative↓/noneΔ)	Sample size, source of information	Authors (ref.)
CIN↑,	IGF-1↑^a^, IGFBP3Δ,	HPVΔ	126 cases (82 CIN), 40 controls	Lee *et al* (61)
LSIL↑^a^, HSIL	IGF-1↑^a^, IGF-1/IGFBP3↑^a^	nd	267 cases, 238 controls	Wu *et al* (62)
CC	IGF-1↑	HPV↑^a^	50 cases, 40 controls	Sharma *et al* (81)
SIL↓	IGFBP-3↑	oncogenic HPV↓		Harris *et al* (78)
SIL↑	IGF-1↑	oncogenic HPV↑	137 cases	
	IGF-1/IGFBP-3	HPV persistence↑		
CCΔ (no prediction)	IGF-1↓, IGFBP-3Δ	nd	72 cases	Huang *et al* (63)
CC, worse OS	IGF-R↑ (predictor)			
HCIN↓	IGF↑, IGFBP-3Δ	HPV-16 and -18↓	329 cases, 621 controls	Schaffer *et al* (64)
CC↑	IGF-1↓^a^, IGF-2↓,	nd	135 cases, 270 controls	Serrano *et al* (65)
HSIL↑ and progression to CC↓	IGFBP-3↓, IGF-R↓	nd	63 cases, 42 controls	Serrano *et al* (66)
HSIL vs. control, LSIL vs CC	IGFBP-3↑^a^, IGFBP-3Δ	HR-HPVΔ	93 cases, 51 controls	Serrano *et al* (68)
CIN III/CC↑	IGF-R↑	nd	cases 90, 30 controls	Kuramoto *et al* (71)
CC↑	IGF-R↑	HPV	72 cases	Huang *et al* (63)
Control vs. all stages of CC↑	IGF-2↑, IGFBP-3↓	nd	160 cases (11 groups), 23 controls	Mathur *et al* (80)
Breast cancer↑	IGF-system components↓	-	72 cases	Voskuil *et al* (72)
Ovarian cancer↓	IGFBP-3↑	nd	59 cases, 108 controls	Dal Maso *et al* (74)
HPV E6/E7 induced cells	IGFBP-3↑ and IGF-1↑	HPV↑	Basic research	Berger *et al* (77)
Cervical cells with E6/E7	IGF-R↑		Basic research	Baege *et al* (76)

For comparison one example of another type of cancer is provided as well as the results of basic research. Details are discussed in the text. IGF, insulin-like growth factor; CIN, cervical intraepithelial neoplasia; HCIN, high-grade CIN; SIL, squamous intraepithelial lesion; HSIL, high-grade SIL; LSIL, low-grade SIL; CC, cervical cancer; OS, overall survival; HR-HPV, high-risk human papillomavirus.

a Statistical significance;

nd, no data.

**Table II:** Examples of therapeutic approaches in IGF and HPV-related cancers.

Target	Method/Tool	Authors (ref.)
IGF-R	IGF-R specific mAbs	Shen *et al* (69), Miller and Yee (97), Hartog *et al* (98), Friedrich *et al* (99)
	Small molecules targeting IGF-1R (tyrosine kinase inhibitors)	Gao *et al* (96), Arcaro (100)
IGF-1 or IGF-2	Ligand-neutralizing antibodies	Gao *et al* (96)
IGF-1R mRNA	Antisense RNA	Nakamura *et al* (105)
	MicroRNA (miR-497)	Luo *et al* (108)
Multiple targets for IGF axis	Multiple drug therapies	Baserga (104), Arcaro (100), Gao *et al* (96)
HPV E6/E7	Synthetic interference RNA	Butz *et al* (110), Hall and Alexander (111)
	Vector-borne siRNA	Gu *et al* (113), Zhou *et al* (115)
	shRNA via VLPs	Bousarghin *et al* (116)
Immune system	Prophylactic vaccines containing VLPs (neutralizing Abs against HPV-L1)	Markowitz *et al* (118)
	Therapeutic vaccines (inducing cytotoxic T lymphocytes )	Berraondo *et al* (121), Peng *et al* (122), Da Silva *et al* (126)
